# Pt- and Pd-decorated MWCNTs for vapour and gas detection at room temperature

**DOI:** 10.3762/bjnano.6.95

**Published:** 2015-04-09

**Authors:** Hamdi Baccar, Atef Thamri, Pierrick Clément, Eduard Llobet, Adnane Abdelghani

**Affiliations:** 1 Carthage University, National Institute of Applied Science and Technology, Nanotechnology Group, Bp676, Centre Urbain Nord, 1080 Charguia Cedex, Tunisia; 2 Carthage University, Unité de Recherche de Synthèse et Structure de Nanomatériaux (UR11ES30), Faculté des Sciences de Bizerte, Jarzouna, 7021, Tunisia; 3 MINOS-EMaS, Universitat Rovira i Virgili, Avda. Països Catalans 26, 43007 Tarragona, Spain

**Keywords:** gas and vapour sensing, metal decoration, mutiwalled carbon nanotubes, plasma treatment, sputtering

## Abstract

Here we report on the gas sensing properties of multiwalled carbon nanotubes decorated with sputtered Pt or Pd nanoparticles. Sputtering allows for an oxygen plasma treatment that removes amorphous carbon from the surface of the carbon nanotubes and creates oxygenated surface defects in which metal nanoparticles nucleate within a few minutes. The decoration with the 2 nm Pt or the 3 nm Pd nanoparticles is very homogeneous. This procedure is performed at the device level (i.e., for carbon nanotubes deposited onto sensor substrates) for many devices in one batch, which illustrates the scalability for the mass production of affordable nanosensors. The response to selected aromatic and non-aromatic volatile organic compounds, as well as pollutant gases has been studied. Pt- and Pd-decorated multiwalled carbon nanotubes show a fully reversible response to the non-aromatic volatile organic compounds tested when operated at room temperature. In contrast, these nanomaterials were not responsive to the aromatic compounds studied (measured at concentrations up to 50 ppm). Therefore, these sensors could be useful in a small, battery-operated alarm detector, for example, which is able to discriminate aromatic from non-aromatic volatile organic compounds in ambient.

## Introduction

Volatile organic compounds (VOCs), nitrogen oxides (NO*_x_*) and hydrogen sulphide are among the most dangerous pollutants released each year by industry into the environment [1–3]. Some VOCs have very harmful effects on human and animal health, even at trace levels in ambient. Indeed, some aromatic VOCs such as benzene are known to be carcinogenic [4–7] and there might not be a safe exposure limit to this compound. Thus, there is a need for the development of simple, affordable and wearable, yet accurate detectors that can alert the general population or workers in various industries (e.g., petrochemical, road construction and repair or landfill sites) of the presence of abnormally high levels of a given pollutant [8–9]. Carbon nanotubes have a high potential to become a gas sensitive material in such detectors, given that they can now be produced at relatively low cost [10–14] with a wide range of structures that can be used for different sensing applications [15–17]. Moreover, carbon nanotubes can operate at room temperature (in contrast to metal oxides that must be operated at relatively high temperatures [18]) thus enabling the development of low-power sensors [13,19]. This is essential for achieving long-life, battery-operated, wearable detectors. Furthermore, carbon nanotube sensors can be easily miniaturised, which is not the case for electrochemical sensors [20].

Pristine carbon nanotubes are known to weakly interact with VOCs in general and with aromatic VOCs in particular. Therefore, a functionalisation of the carbon nanotube sidewalls is essential to promote sensitivity. In previous works, we used oxygen-plasma-treated multiwalled carbon nanotubes for detecting nitrogen dioxide, ammonia, benzene, toluene, acetone, methanol and ethanol [21–25]. Instead of the typical functionalisation methods, it was possible to decorate the carbon nanotubes with various metal or metal oxide nanoparticles. These nanoparticles may show different reactivity to different chemical species, which affects the sensitivity and selectivity of the hybrid carbon nanotube material. The idea is to use nanoparticles that donate or accept charge upon adsorption of vapours or gas molecules, which eventually alters the electron transport in the carbon nanotube [26]. Kumar et al. published the first application of this concept by producing platinum-decorated carbon nanotubes by means of a wet-chemistry technique [27]. Star et al. electroplated carbon nanotubes with gold, rhodium, palladium or platinum to obtain metal-decorated nanotubes as gas sensors for carbon monoxide, nitrogen dioxide, methane, hydrogen sulphide, ammonia and hydrogen [28]. However, this technique leads to an inhomogeneous decoration of the carbon nanotubes with high irregularities in the shape and size of the metal nanoparticles, which eventually may result in poor sensor reproducibility. Espinosa and co-workers decorated plasma-treated carbon nanotubes with Au or Ag using an evaporation technique for NO_2_ detection at room temperature [29]; however, their sensors were not fully reversible. Penza and co-workers decorated CVD grown carbon nanotubes with Au, Pt or Pd by using sputtering to enhance sensor response towards NO_2_ and NH_3_ [30–31]. However, the response was substantial only at an operating temperature of 200 °C, which is well above room temperature. Guo and co-workers oxidised carbon nanotubes in nitric/sulfuric acid and decorated them with Pd for detecting benzene [32]. Additionally, Lu and co-workers employed Pd-coated carbon nanotubes for discriminating NO_2_, HCN, HCl, Cl_2_, acetone and benzene [33]. The sensors from these two groups showed low sensitivity [32–33] at concentrations three orders of magnitude higher than those required for environmental protection applications. Leghrib and co-workers introduced multiwalled carbon nanotubes decorated with Au, Pd or Ni nanoparticles (formed by evaporation of metals) and also with Rh or Pt nanoparticles (from a colloidal solution) for detecting benzene, nitrogen dioxide and hydrogen sulphide [34–36]. Although the detection could be performed at room temperature, recovering the sensor baseline required heating at 150 °C. This heat treatment to regain the sensor baseline was also observed by Mudimela and co-workers when they used vertically aligned carbon nanotubes decorated with sputtered Au nanoparticles to detect nitrogen dioxide [37]. Finally, Clément and co-workers reported the use of an organometallic precursor together with a plasma treatment in order to obtain FeO-decorated multiwalled carbon nanotubes for the room-temperature detection of aromatic and non-aromatic VOCs [38]. In spite of the fact that metal decoration effectively helps in tuning the gas/vapour response of carbon nanotubes, selectivity remains an important challenge that is still far from being achieved.

In the quest to improve selectivity, in this paper, we report on the gas sensing properties of carbon nanotubes decorated with sputtered Pt or Pd nanoparticles. Sputtering is an advantageous technique for functionalising carbon nanotubes since it allows for an oxygen plasma treatment followed by metal decoration without breaking the vacuum or the need to transfer the nanotubes between different reactors and additionally takes only a few minutes. Furthermore, this treatment can be performed at the device level (i.e., for carbon nanotubes deposited onto sensor substrates) and allows for many devices to be processed in one batch. Such advantages make this approach very interesting for the mass production of affordable nanosensors. The response to both aromatic (toluene and benzene) and non-aromatic (ethanol, methanol and acetone) VOCs together with the response to a pollutant gas (NO_2_) have been investigated.

## Experimental

### Carbon nanotube synthesis, functionalisation and metal decoration

The carbon nanotubes used in the experiment were purchased from Nanocyl s.a. (Belgium). They were synthesised by catalytic chemical vapour deposition and their purity was higher than 95% (Nanocyl^TM^ NC3100). These multiwalled carbon nanotubes (MWCNT) were up to 50 μm in length and their outer and inner diameters ranged from 3–15 nm and 3–7 nm, respectively. The MWCNTs were dispersed in *N*,*N*-dimethylformamide, ultrasonically stirred for 20 min at room temperature (200 W Ultrasonic Bath, Selecta S.A., Spain), and subsequently airbrushed (JB1113N automatic dispenser and nozzle, Fisnar, Inc., USA) onto Au comb electrodes (electrode gap was 500 μm) screen-printed on alumina substrates. The resistance of the resulting film was monitored during the deposition, enabling the production of sensors with reproducible baseline values [25,38]. During deposition, the substrates were kept at 100 °C, which ensured a fast evaporation of the solvent and good adhesion of the carbon nanotubes to the substrate. This temperature ensures the complete evaporation of the solvent in which carbon nanotubes are dispersed upon reaching the heated substrate. A lower temperature would result in solvent wetting of the substrate during the deposition, and a higher temperature would result in the solvent being totally evaporated before the nanotubes actually reach the substrate. These two situations are undesirable since the former leads to a non-uniform deposition and the latter to poor adhesion of the films. The MWCNT-coated sensor substrates were placed inside the chamber of an ATC Orion-8-HV multitarget sputtering machine (AJA International, Inc., USA). In the first step, an oxygen plasma treatment was performed at a pressure of 0.1 Torr using a power of 15 W for 1 min. An inductively coupled plasma at a frequency of 13.56 MHz was used during this process. A controlled ﬂow of oxygen and argon was introduced into the chamber, which gave rise to functional oxygen species attached to the carbon nanotube sidewalls [24]. These controlled oxygenated defects are known to act as nucleation sites for metal nanoparticles [39]. In the second step, plasma-treated MWCNT sensors were decorated with either Pt or Pd nanoparticles. For Pt, the sputtering parameters were adjusted to 15 W for 10 s, and for the Pd power to 150 W for 8 s. In both cases, the pressure was kept at 3.75 mTorr and a controlled ﬂow of argon was introduced into the chamber. Pt and Pd targets with purity greater than 99.95% were employed.

### Morphology and composition studies

The morphology and composition of sputtered, metal-decorated CNTs were studied by transmission electron microscopy (TEM) and by X-ray photoelectron spectroscopy (XPS), respectively. For the TEM analysis, pristine MWCNTs were dropped onto a commercial, lacey-carbon grid. This was then placed inside the sputtering chamber and underwent the plasma treatment and metal decoration steps at the same time that the sensors were prepared. TEM experiments were carried out in a JEOL model 1011 system operating at 100 kV. The chemical composition of the samples was studied using XPS. A Versaprobe PHI 5000 spectrometer from Physical Electronics, equipped with a monochromatic Al Kα X-ray source with 0.7 eV energy resolution was employed. To avoid sample surface charging during the experiment, a dual-beam charge neutralisation comprised of an electron gun of 1 eV and an argon ion gun (≤10 eV) was used as reported in [40].

### Gas sensing measurements

The electrical characterisation under gas or vapour environment was performed employing an HP 4192A impedance analyzer [17]. Since all devices showed a resistive behavior at frequencies below 100 kHz, the resistance of the metal-decorated carbon nanotube films in the presence of different VOCs or gases was studied by employing a fixed operating frequency of 1 kHz [24]. This facilitated the direct, real-time measurement of resistance response signals. Dry air was used as a carrier gas and as a balance gas. Two mass flow meters and a thermostated bubbler were used to generate reproducible concentrations of the different volatile compounds tested. Calibrated gas bottles connected to the mass flow meters were used to generate reproducible concentrations of the gas studied. These were coupled to a stainless steel sensor chamber (35 cm^3^ volume), which housed two carbon nanotube sensors. More details on this setup can be found in [24]. A constant flow of 100 sccm was used throughout the measurements (both during the response and cleaning phases). Unless otherwise specified, the sensors were operated at room temperature (25 °C) and the moisture level within the sensor chamber was 10% RH.

## Results and Discussion

### Morphological and compositional studies

**Figure 1:**
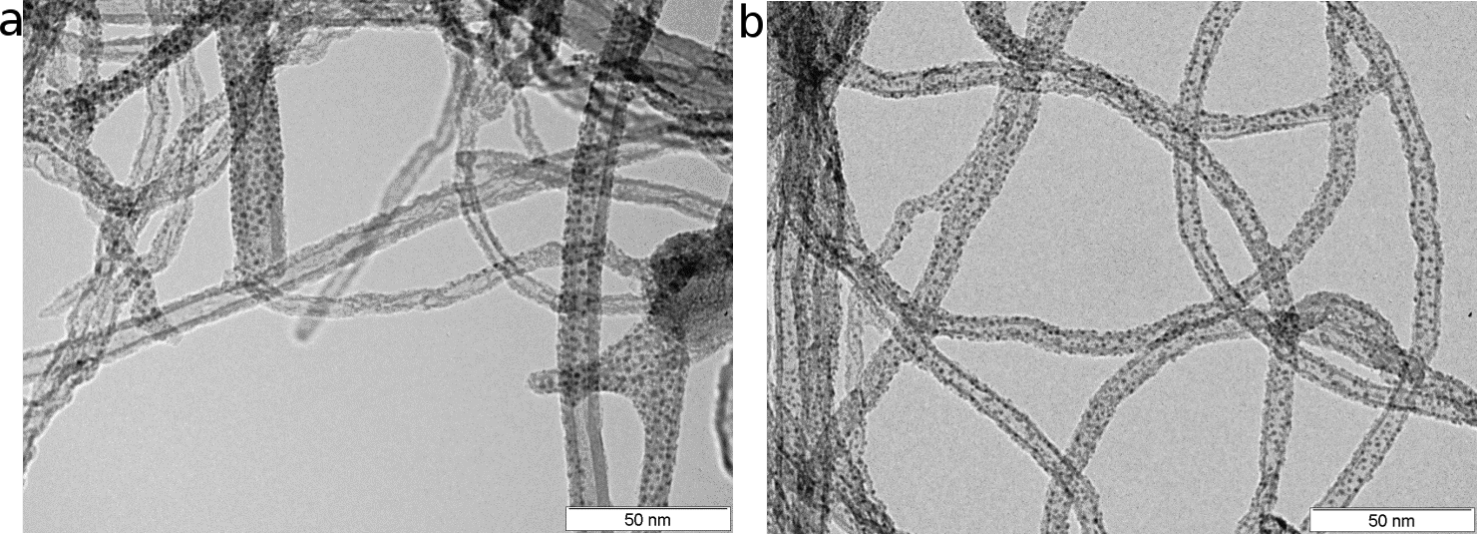
TEM images of the Pd-decorated MWCNTs (a) and Pt-decorated MWCNTs (b) resulting from the rf sputtering treatment.

The morphology of the plasma-treated, metal-decorated, multiwalled carbon nanotubes was studied by TEM and the main results are summarised in Figure 1. It can be derived that the sputtering technique employed herein leads to a very uniform decoration of the external wall of the MWCNTs when using either Pd or Pt nanoparticles. The average size of the Pd nanoparticles is slightly larger than 3 nm, while the size of Pt nanoparticles is about 2 nm. The homogeneity of the decoration and the small nanoparticle diameter achieved by sputtering is clearly superior to that of our previously reported results in which different approaches for nanotube decoration were implemented [34,36,38].

**Figure 2:**
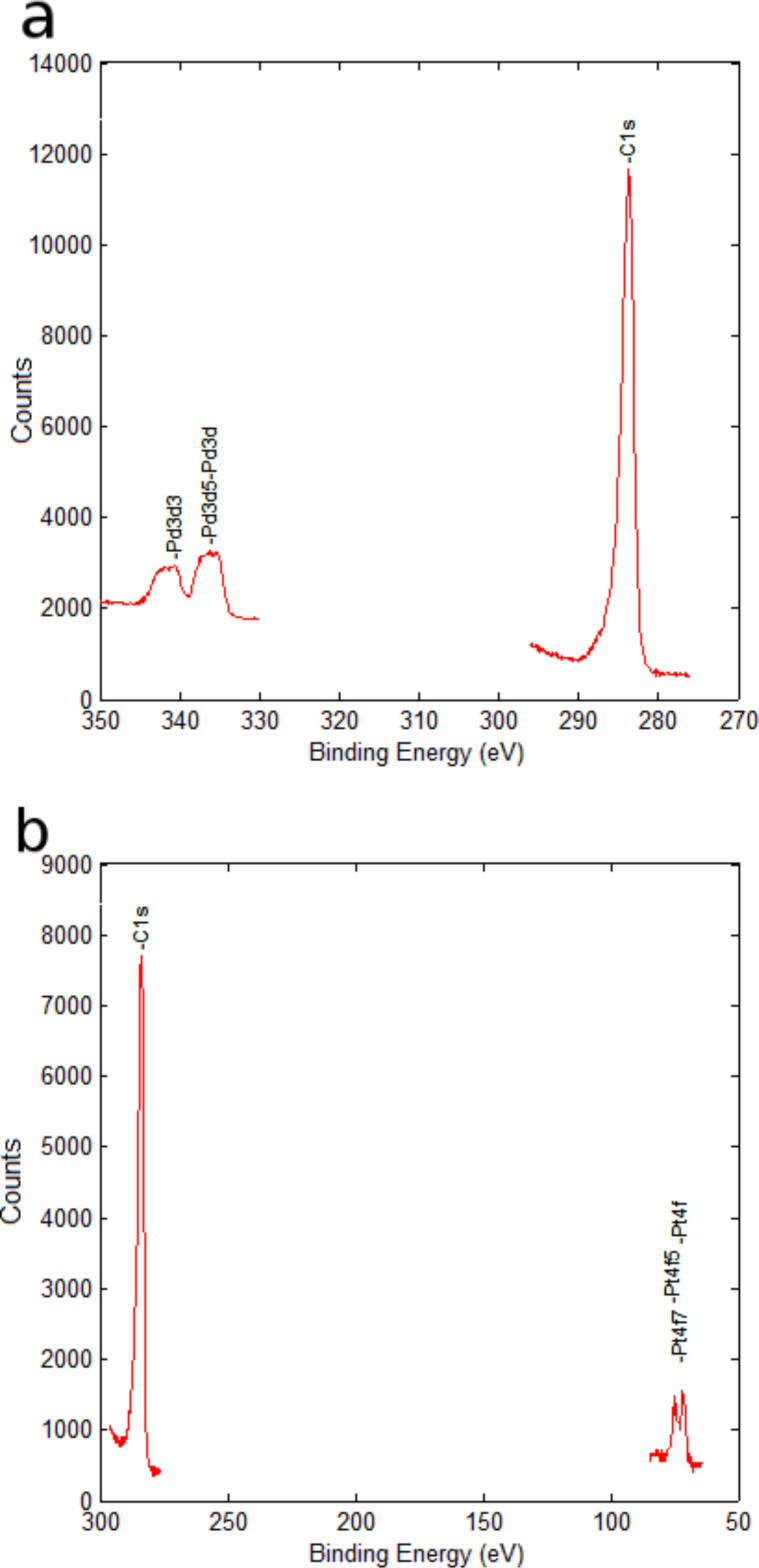
High-resolution XPS spectra in the C 1s and Pd 3d regions of a Pd–MWCNT sensor (top) and the C 1s and Pt 4f regions of a Pt–MWCNT sensor (bottom).

The composition of oxygen-plasma-treated MWCNT films was studied in detail using XPS and the results were reported in [23–25]. The analysis reveals that the oxygen plasma treatment results in hydroxyl, carbonyl and carboxyl functionalities being added to the surface of carbon nanotubes. Carbonyl functions are, by far, the majority among those added. The presence of Pt or Pd on the surface of decorated MWCNTs was confirmed by XPS. Figure 2 shows the high resolution spectra in the C 1s and Pd 3d regions and the C 1s and Pt 4f regions of a Pd–MWCNT sensor and a Pt–MWCNT sensor, respectively. The elemental composition determined by this technique indicated that the amounts of Pt or Pd were near 2 atom % (see Table 1).

**Table 1:** Elemental chemical composition percentage as derived from an XPS analysis of the Pt-decorated and Pd-decorated carbon nanotube samples.

	Relative composition (atom %)				
	C	O	Pt	Pd	Na

Pt–MWCNT	83.69	14.56	1.75	–	<1
Pd–MWCNT	83.77	14.18	–	2.05	<1

### Sensing results

#### Detection of aromatic and non-aromatic VOCs

**Figure 3:**
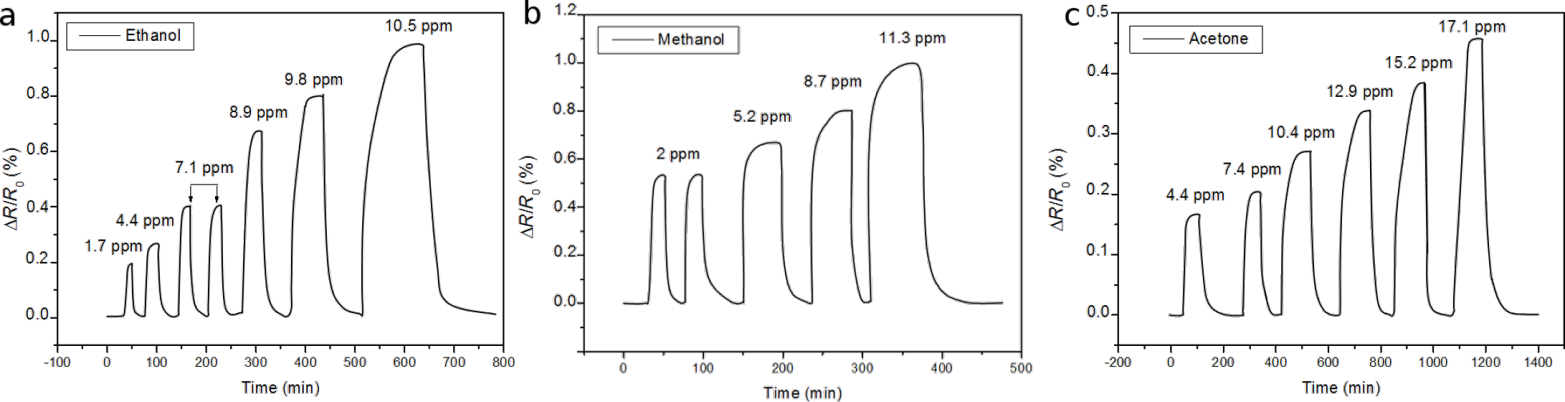
Pt-decorated MWCNTs sensor response to (a) ethanol, (b) methanol and (c) acetone vapours.

**Figure 4:**
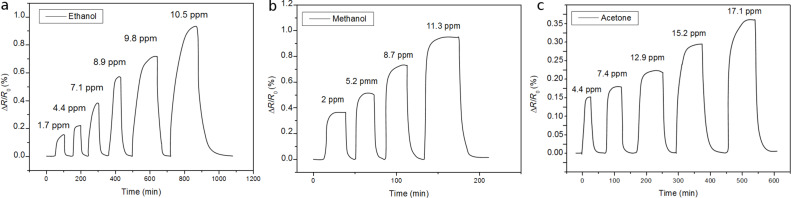
Pd-decorated MWCNTs sensor response to (a) ethanol, (b) methanol and (c) acetone vapours.

The variation in the resistance of MWCNTs decorated with Pd or Pt in the presence of different concentrations of non-aromatic and aromatic VOCs was studied. The sensors were operated at room temperature. The sensor response was defined as the normalised resistance change defined by:

[1]
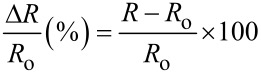


where *R* is the resistance of a MWCNT film measured under a given vapour and *R*_o_ is the baseline resistance of the film (i.e., resistance under clean air). The Pt- or Pd-decorated MWCNT sensors were not responsive to the aromatic VOCs tested (i.e., benzene and toluene). High concentration levels of these compounds were tested (up to 50 ppm) without any significant response. In that sense, Pt- or Pd-decorated MWCNTs behave similarly to Au-decorated MWCNTs. According to a theoretical study, the interaction between benzene and Au–carbon nanotubes consists of a weak physisorption, as shown by the large bond length (≈3.9 Å) and a low binding energy (0.193 eV), with no significant charge transfer between the adsorbed molecule and the Au–carbon nanotube system [26]. This indicates that Au-decorated carbon nanotubes are not suitable for detecting benzene. This is in contrast with bare oxygen-plasma-treated or Rh- and FeO-decorated MWCNTs, which were found to be responsive to aromatic VOCs [24,36,38]. Pd- and Pt-decorated MWCNT sensors were responsive to the non-aromatic VOCs tested. Typical response and recovery cycles toward non-aromatic VOCs are shown Figure 3 and Figure 4.

The sensor resistance increased for increasing concentrations of these compounds. Plasma-treated and metal-decorated MWCNT behave generally as mild p-type semiconductors [26,34] and the non-aromatic VOCs tested are reducing species. Upon adsorption, electronic charge is transferred from the molecule to the metal nanoparticle–MWCNT system, which results in a decrease in the conductance of the nanotube.

**Figure 5:**
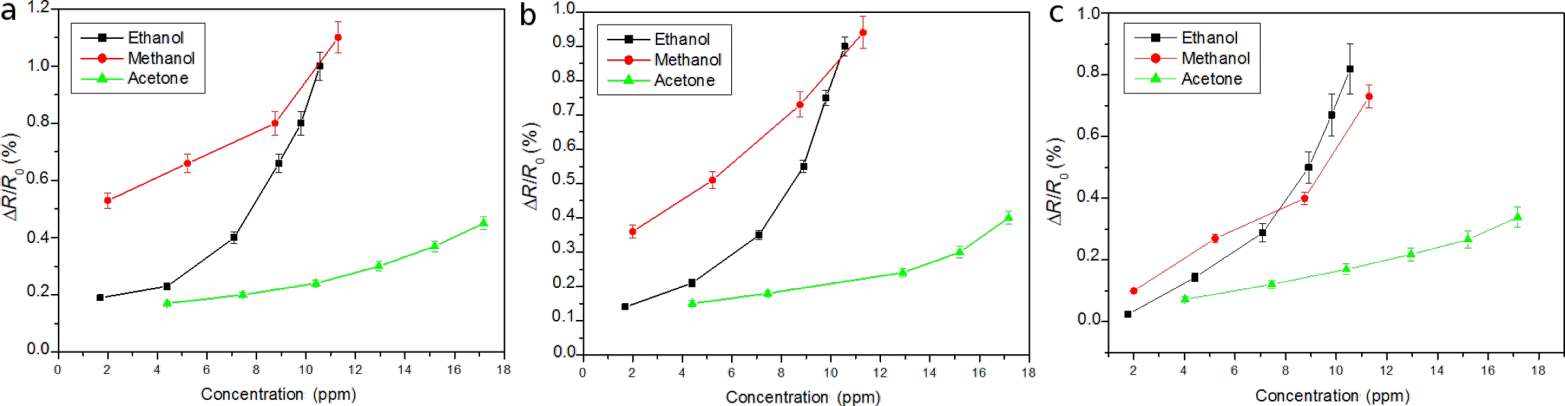
(a) Calibration curves of Pt–MWCNT sensors to vapours. (b) Calibration curves of Pd–MWCNT sensors to vapours. (c) Calibration curves for oxygen-plasma-treated, bare MWCNT sensors to vapours.

The lower concentrations shown in Figure 3 and Figure 4 are far from the limit of detection for these compounds, considering that a clear response signal should be at least three times higher than the noise level. In general, Pt-decorated MWCNTs showed higher response to non-aromatic VOCs than Pd-decorated MWCNTs. The work function of oxygen-plasma-treated MWCNTs ranges from 4.9 to 5.1 eV [41]. This is very close to values of metals such as Pt (4.8 eV) or Pd (4.95 eV) [34], enabling electrons to pass easily between the metal nanoparticles and CNTs. The direction of the charge transfer depends on the composition of the surrounding gas. Furthermore, the electronegativity values for Pt and Pd are quite similar and so is the relative weight (in atom %) of the metal dopants in both types of MWCNT samples. Therefore, the differences that arise in response may be due to the two following reasons. First, the diameter of nanoparticles is slightly different, with the Pt nanoparticles being slightly smaller than the Pd nanoparticles. A small diameter, corresponding to a larger overall surface area, is essential to maximize the effect of adsorbates on metal clusters, which may result in higher sensitivity. Second, while the duration of the sputtering decoration step was basically the same for Pt and Pd, the power used for decorating MWCNTs with Pd was significantly higher than for Pt. Therefore, this may lead to a more defective surface for Pd-decorated MWCNTs and result in a degradation of the electronic properties of nanotubes. The calibration curves for non-aromatic vapours are shown in Figure 5.

According to the results shown in Figure 5, the response of Pd- and Pt-decorated sensors to methanol and ethanol is clearly higher (especially at the lower concentrations tested) than that of bare carbon nanotube sensors. This implies that the limit of detection for such species would be lower for Pt- or Pd-decorated carbon nanotube sensors than for bare carbon nanotube sensors. In contrast, the response to acetone is basically the same. Furthermore, oxygen-plasma-treated, bare carbon nanotube sensors have been found to be responsive to aromatic VOCs [24], which is not the case for the Pt- or Pd-decorated carbon nanotube sensors reported here. These important changes in the response patterns of Pt- or Pd-decorated carbon nanotubes compared to those of bare carbon nanotubes could help to increase selectivity if integrated into a sensor array.

#### Detection of nitrogen dioxide

**Figure 6:**
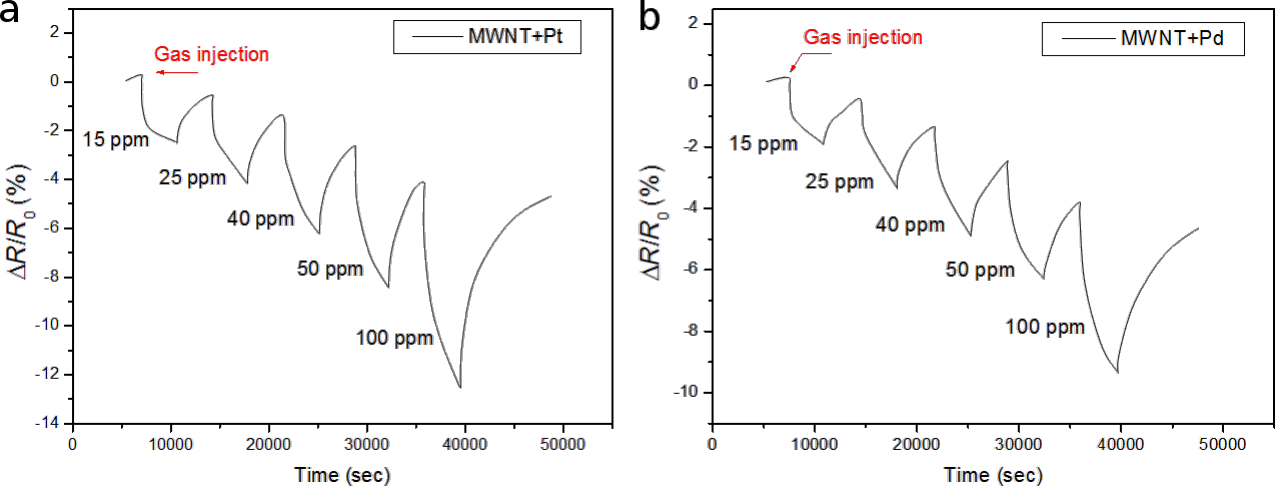
(a) Response of a Pt- decorated MWCNTs sensor to various concentrations of NO_2_. (b) Response of Pd-decorated MWCNTs sensor to various concentrations of NO_2_.

The sensors decorated with Pd and Pt nanoparticles were tested for different concentrations against NO_2_. The sensors were again operated at room temperature. Figure 6 shows the typical response and recovery cycles for increasing concentrations of NO_2_ for Pt- and Pd-decorated MWCNTs. The sensor response was calculated according to Equation 1 and is negative because the resistance decreased in the presence of nitrogen dioxide.

Again, the Pt-decorated MWCNT sensors are more responsive to nitrogen dioxide than Pd–MWCNT sensors. The sensors are very responsive to NO_2_ and their limit of detection is lower than the lowest nitrogen dioxide concentration tested. In fact, the binding energy of nitrogen dioxide molecules to the metal nanoparticle–carbon nanotube system is rather strong, since the full recovery of the baseline resistance of the sensors is not reached, and a significant drift appears in the successive detection/recovery events shown in Figure 6. Nitrogen dioxide strongly binds to the surface of metal-decorated carbon nanotubes and does not completely desorb during the cleaning phase. For the full recovery of the baseline, either the cleaning period at room temperature should be significantly extended, or low temperature heating (i.e., 150 °C) should be applied in short intervals to promote the desorption of nitrogen dioxide from the surface of the active nanomaterial. Similar results were previously observed for the detection of this pollutant gas with different types of metal-decorated MWCNT sensors [34–36].

**Figure 7:**
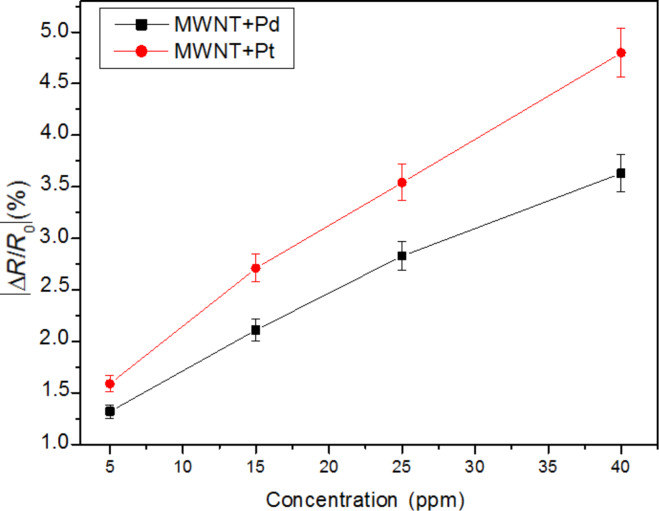
Calibration curves of Pd– MWCNT and Pt–MWCNT sensors to NO_2_.

Figure 7 shows the calibration curves for the detection of NO_2_ using Pd- and Pt-decorated MWCNTs. Considering the slope of the calibration curves, it can be derived that the sensitivity toward nitrogen dioxide for Pt–MWCNTs is slightly better than that of Pd–MWCNTs.

**Figure 8:**
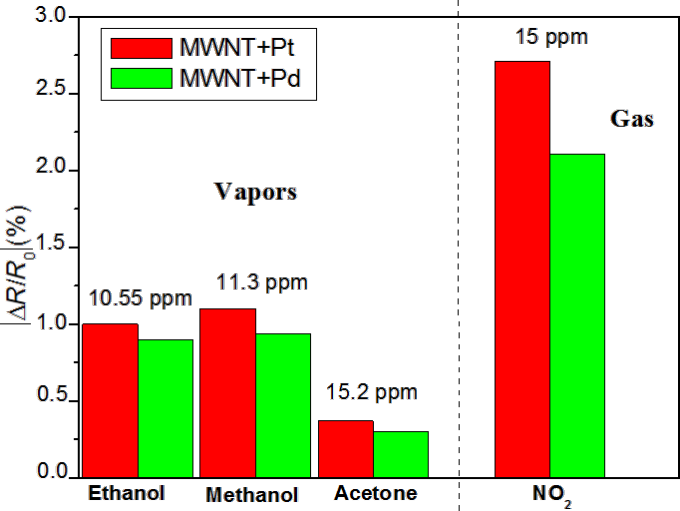
Comparison between the response of Pd–MWCNTs and Pt–MWCNTs to the different gases and vapours tested.

Figure 8 summarises the responses of the two types of sensors for the different vapours and gases tested. The most important result is that the two different nanomaterials tested are not responsive to aromatic VOCs. This is useful for the development of sensor arrays with differently decorated, multiwalled carbon nanotubes resulting in detection systems with significantly improved selectivity.

The mean sensitivity (mean slope of the calibration curve calculated over the whole concentration range) for the Pd- and Pt- decorated carbon nanotube sensors and for a bare carbon nanotube sensor was computed for the different species measured. These results are summarised in Table 2. The decoration with Pt or Pd results in a 40% increase in sensitivity to ethanol (compared to bare carbon nanotubes) and also a moderate increase in sensitivity towards methanol and acetone. In contrast, the Pt- and Pd-decorated MWCNT sensors show a significant decrease in sensitivity to nitrogen dioxide as compared to the sensitivity of bare MWCNT sensors. However, to our knowledge, this is the first time that the response of Pd- or Pt-decorated carbon nanotubes toward methanol, ethanol and acetone is reported, although other authors have studied the response of such hybrid nanomaterials toward nitrogen dioxide. Star and co-workers report a small response of a Pd-decorated single-walled carbon nanotube sensor to NO_2_ in [28]; however, enough data is not available to estimate the sensitivity. Penza and co-workers have reported nitrogen dioxide sensitivities of about 80 ∙ 10^−2^ % ppm^−1^ both for Pt- and Pd-decorated MWCNTs operated at 200 °C [31]. Although this sensitivity is high, the sensors were operated at a relatively elevated temperature. Finally, Leghrib and co-workers reported a nitrogen dioxide sensitivity of about 45 ∙ 10^−2^ % ppm^−1^ for a Pd–MWCNT sensor operated at room temperature [34]. In that case, Pd nanoparticles were formed by evaporation onto carbon nanotubes and this sensor was also somewhat responsive to aromatic VOCs.

**Table 2:** Mean values of the sensitivity (10^−2^ % ppm^−1^) to the different vapours and gasses tested for Pt–MWCNT, Pd–MWCNT and bare MWCNT sensors.

	Ethanol	Methanol	Acetone	Nitrogen dioxide

Pt–MWCNT	9.9	6.7	2.1	9.4
Pd–MWCNT	9.5	6.7	1.9	6.9
Bare MWCNTs	7.1	6.3	1.7	17.8^a^

^a^Sensitivity computed from data reported in [22].

## Conclusion

In this paper, we have reported on the gas sensing properties of carbon nanotubes decorated with Pt or Pd nanoparticles. Nanoparticles were formed by rf sputtering, which allowed an oxygen plasma treatment directly followed by the metal decoration step without breaking the vacuum or the need to transfer the nanotubes between different reactors. This is highly advantageous for scaling up the process for the production of gas-sensitive nanomaterials in an efficient and cost effective way. The response to both aromatic (toluene and benzene) and non-aromatic (ethanol, methanol and acetone) VOCs together with the response to a pollutant gas such as NO_2_ have been investigated. None of the sensors tested were responsive towards benzene or toluene vapours. In contrast, both the Pt- and Pd-decorated MWCNTs were responsive to the non-aromatic VOCs when operated at room temperature. The Pt–MWCNT sensors were more responsive, which was attributed to the fact that the Pd sputtering process was more aggressive towards the carbon nanotubes (i.e., the sputtering of Pd involved using a significantly higher power than Pt). However, both types of sensors were also responsive to nitrogen dioxide, as was previously observed for Au-, Ni- or Rh-decorated MWCNT sensors.

Considering that in previous works it was possible to detect both aromatic and non-aromatic VOCs employing bare, oxygen-plasma-treated MWCNTs and FeO-decorated MWCNTs, the sensors studied here are of interest since they are unresponsive to aromatic VOCs. Therefore, by combining, for example, a Pt–MWCNT sensor with FeO-decorated or oxygen-treated MWCNT sensor in an array, and by applying standard pattern recognition methods, it would be possible to determine the presence of aromatic VOCs in a background in which the presence of non-aromatic VOCs is likely to occur.
